# Influence of Irradiance and Temperature on the Virus MpoV-45T Infecting the Arctic Picophytoplankter *Micromonas polaris*

**DOI:** 10.3390/v10120676

**Published:** 2018-11-29

**Authors:** Gonçalo J. Piedade, Ella M. Wesdorp, Elena Montenegro-Borbolla, Douwe S. Maat, Corina P. D. Brussaard

**Affiliations:** 1 Department of Marine Microbiology and Biogeochemistry, NIOZ Royal Netherlands Institute for Sea Research, and University of Utrecht, P.O. Box 59, 1790 AB Den Burg, Texel, The Netherlands; goncalo.piedade@nioz.nl (G.J.P.); Ella.wesdorp@nioz.nl (E.M.W.); elenamnbr@gmail.com (E.M.-B.); douwe.maat@nioz.nl (D.S.M.); 2 Aquatic Microbiology, Institute for Biodiversity and Ecosystem Dynamics, University of Amsterdam, P.O. Box 94248, 1090 GE Amsterdam, The Netherlands; 3 Department of Systems Biology, Spanish National Center for Biotechnology (CNB), Calle Darwin, 3, 28049 Madrid, Spain

**Keywords:** Arctic algal viruses, Light intensity, Light regime, Global Climate Change, Virus growth characteristics

## Abstract

Arctic marine ecosystems are currently undergoing rapid changes in temperature and light availability. Picophytoplankton, such as *Micromonas polaris*, are predicted to benefit from such changes. However, little is known about how these environmental changes affect the viruses that exert a strong mortality pressure on these small but omnipresent algae. Here we report on one-step infection experiments, combined with measurements of host physiology and viability, with 2 strains of *M. polaris* and the virus MpoV-45T under 3 light intensities (5, 60 and 160 μmol quanta m^−2^ s^−1^), 2 light period regimes (16:8 and 24:0 h light:dark cycle) and 2 temperatures (3 and 7 °C). Our results show that low light intensity (16:8 h light:dark) delayed the decline in photosynthetic efficiency and cell lysis, while decreasing burst size by 46%. In contrast, continuous light (24:0 h light:dark) shortened the latent period by 5 h for all light intensities, and even increased the maximum virus production rate and burst size under low light (by 157 and 69%, respectively). Higher temperature (7 °C vs 3 °C) led to earlier cell lysis and increased burst size (by 19%), except for the low light conditions. These findings demonstrate the ecological importance of light in combination with temperature as a controlling factor for Arctic phytoplankton host and virus dynamics seasonally, even more so in the light of global warming.

## 1. Introduction

Viruses are important mortality agents of phytoplankton, exerting a substantial top-down control on their host populations [1,2,3,4,5,6,7]. They act as key modulators of the microbial food web and as such, biogeochemical cycling and trophic efficiency [8,9]. Environmental factors such as nutrients, temperature and light have been shown to affect algal host–virus interactions, yet the number of studies is still limited [10]. This gap in knowledge precludes not only insights into how regular seasonal changes impact virus-induced phytoplankton host population dynamics and community structure, but also to what extent the predicted global climate change-induced alterations in physicochemical variables impact the ecological role of marine viruses.

Polar regions, such as the Arctic, are subjected to strong seasonal variations in light, temperature, and salinity. The Arctic is reported to be one of the fastest warming areas on Earth, with summer sea surface temperatures in ice-free regions ranging from 0 to as high as 11 °C [11,12] with a predicted rise of 0.03–0.05 °C per year in the 21st century [13]. The Arctic productive season is short and its onset is largely regulated by light availability [14], which is expected to change as a result of global climate change [15,16]. Indeed, reduced ice cover, earlier and strengthened salinity-induced vertical stratification of the upper water column, increased sediment input from melting glaciers and increased coastal erosion and river flow will affect light availability in Arctic coastal waters [13,17].

The stronger salinity induced stratification of the upper water column will also stop the flux of nutrient rich waters to the surface, inducing a nutrient limitation of the surface layers [17], which has been found in turn to promote pico-sized phytoplankton in the Arctic [18,19]. Additionally, ongoing pCO_2_ enhancement seems to stimulate Arctic picoeukaryotic phytoplankton growth, further promoting the shift towards a picophytoplankton dominated Arctic Ocean [20,21].

*Micromonas polaris* is commonly the dominant picoeukaryotic phytoplankton species in the Arctic [22,23,24,25,26]. Maat et al. [27] reported for the first time the isolation of four prasinoviruses (MpoVs) infecting *M. polaris.* To this date, this is the only reported psychrophilic eukaryotic algal host–virus model system brought into culture, presenting an unparalleled opportunity to study the virus–host interactions in response to ecologically relevant environmental variables under controlled lab conditions. Thus far, only the effects of temperature have been studied for this polar algal host–virus model system, whereby increased temperature was found to stimulate the proliferation of MpoV [27]. However, this experiment was executed at only one light intensity (70–90 µmol quanta m^−2^ s^−1^). Irradiance is an important additional ecological variable to study additionally as it strongly varies over the season and interacts with temperature on the level of host physiology [28]. To date, light manipulation experiments have only been performed with temperate (15 °C) *Micromonas* species and their respective viruses MpV-02T and MpV-08T, and did not reveal an effect of light intensity on virus production [29,30]. However, the low light intensity used in these temperate studies was 25 μmol quanta m^−2^ s^−1^, whereas *M. polaris* perseveres and has a competitive advantage over other algae at far lower light intensities [23,24].

Studying how light and temperature affect the host–virus interactions is key to understanding polar ecosystem function and to predicting the effects of global climate change on microbial interactions, including lytic viral infection. Here we studied the influence of light intensity (i.e., 5 compared to 60 and 160 μmol quanta m^−2^ s^−1^), light period (16:8 and 24:0 h light:dark cycle) and temperature (3 and 7 °C) on the virus growth characteristics of MpoV-45T infecting *M. polaris*. We hypothesized that (1) low light intensity negatively affects virus production, (2) a longer light period (24:0 h light:dark) partially counters light limitation effects by providing extra energy during the infection cycle, and (3) higher temperature positively affects algal growth rate and in turn virus growth characteristics. The informative power of one-step infection experiments (i.e., latent period, virus production rate, and burst size) was combined with measurements of photosynthetic efficiency, cell characteristics (red fluorescence and forward and side scatter), intracellular reactive oxygen species, and percentage of dead cells.

## 2. Materials and Methods

### 2.1. Species and Culturing Conditions

For this study the Arctic picoeukaryotic photoautotroph *Micromonas polaris* strains RCC2257 and RCC2258 (Roscoff Culture Collection) were used in combination with the Arctic lytic virus MpoV-45T (NIOZ culture collection; [27]). To test for strain-specific interactions we selected strains from the same geographic area and good infection performance [27]. Both *M. polaris* strains were cultured in Mix-TX medium [27] at 3 °C and kept in exponential growth phase by regular transfer to new medium. The cultures were adapted to different photosynthetically active radiation (PAR) light intensities, i.e., 5, 60 and 160 µmol quanta m^−2^ s^−1^ (respectively, low, mid, and high light: LL, ML, and HL) under a 16:8 h light:dark (L:D) cycle for more than 3 months. Moreover, *M. polaris* RCC2258 was also adapted (for 3 months) to continuous PAR irradiance 24:0 h L:D (at 3 °C), and to 7 °C (with 16:8 h L:D cycle). The incubation cabinets were equipped with Panasonic 40 FL (40SS ENW/37) or UO 20W/ 865 T8 ROT lamps. The type of lamp did not affect the algal growth differently.

Maximum algal growth rates (d^−1^; Table 1) under the different experimental conditions were derived (by fitting an exponential growth model) from the temporal dynamics of exponentially growing phytoplankton in dilute cultures (1000–10,000 cells starting concentration and counted by flow cytometry [31]).

*M. polaris* RCC2258 cultured at ML and 3 °C was used as the standard host for maintaining MpoV-45T. Viral lysates were maintained by regularly transfer to exponentially growing host (10% v/v). Full lysis of the algal host culture was determined upon visual screening (clearing of the cultures).

### 2.2. One-Step Viral Infection Experiments

Once the algal hosts were acclimatized to the different culture conditions, one-step infection experiments were conducted in duplicate with 200 mL exponentially growing algae (appr. 5 × 10^5^ mL^−1^) and freshly produced MpoV-45T lysate at a virus to host cell ratio between 13–35 (on average 24). Two non-infected controls received Mix-TX medium with the same volume as the lysate for the infected cultures. Upon infection, and throughout the infection cycle, samples (7 mL) were taken for flow cytometric characterization and enumeration of the algal host and virus, host photosynthetic efficiency (Fv/Fm), cellular reactive oxygen species (ROS), and percentage of dead algal cells (SYTOX Green stained). Samples were directly analyzed except for virus enumeration samples, which were fixed with glutaraldehyde (25% glutaraldehyde, EM-grade, Sigma-Aldrich, USA) to a final concentration of 0.5% (v/v), flash frozen in liquid nitrogen and stored at –80 °C until analysis. The first sampling (T0) was performed immediately after the addition of the viruses to the cultures and further samples were taken every 5 h for the first 35 h of the experiment, twice a day during the third and fourth day of the experiment and once a day onwards until the algal cultures were fully lysed.

The number of infectious MpoV-45T used and produced during the experiments was determined by end-point dilution using exponentially growing *M. polaris* RCC2258 (12 ten-fold dilutions, *n* = 5) [32]. Infected cultures were visually screened for clearance of the algal cultures twice a week (max 3 weeks). Virus infectivity was calculated using the MPN analyzer [33]. The percentage of infective viruses was calculated by dividing the number of infective viruses (MPN) by the number of total viruses as determined by flow cytometry [34].

### 2.3. Photosynthetic Efficiency

Pulse amplitude modulated (PAM) fluorometry was used to determine the maximal photosystem II photosynthetic efficiency of the algal host cultures [35]. Measurements were conducted using a Walz PAM-Control coupled with a Walz WATER-ED Emitter-Detector-Unit and Mix-TX media was used as blank. After dark adaptation (1 h on ice) the algal samples (3.5 mL) were gently poured into a Water-K quartz cuvette and minimum fluorescence (F0) was measured. After a saturation pulse the maximum fluorescence (Fm) and photosynthetic efficiency (Fv/Fm in relative units, r.u.) were determined, whereby the variable fluorescence (Fv) is defined as Fm–F0. We defined the drop in photosynthetic capacity in the infected cultures as the time that Fv/Fm decreased consistently below 10% of the starting ratio.

### 2.4. Flow Cytometry

Phytoplankton were enumerated on fresh samples using a Beckton Dickinson Accuri C6 flow cytometer equipped with a 488 nm argon laser, with the trigger set to chlorophyll red autofluorescence [31]. Phytoplankton abundances were normalized to the initial abundance to allow for optimal comparison between experiments with occassionally sliglty diferent starting concentrations. Cellular flow cytometric characteristics of *Micromonas*, i.e., red fluorescence (RFL), forward scatter (FSC; measure of cell size) and 90° side scatter (SSC, measure of cell granularity) [36,37], were extracted as geometric means of the population. The start of host lysis was defined as the time where >5% decrease in cell abundances was detected. Full lysis of the host culture was defined as the time where >95% of the cells lysed. The percentage of dead algal cells was determined by SYTOX-Green (ThermoFisher Scientific, Waltham, EUA) staining according to Brussaard et al. [38]. Briefly, SYTOX was added to fresh subsamples (final concentration 0.5 μM) and incubated in the dark for 15 min, after which cells were analyzed using flow cytometry (trigger set on RFL). Per treatment fresh uninfected and formaldehyde-killed samples served as negative and positive control for gate setting. The percentage of dead cells was calculated by dividing the number of stained cells by the total algal abundance.

The reactive oxygen species (ROS) relative content of the 16:8 h L:D cultures at 3 °C was determined according to Evans et al. [39]. In short, the green fluorescent dye 2’,7’-dichlorodihydrofluorescein diacetate (H_2_DCFDA) was used to a final concentration of 10 µM. Samples were incubated for 1 h at culture conditions and measured using flow cytometry. The relative ROS content was extracted from the geometric mean of the cellular green fluorescence (GFL) of the population.

The abundances of MpoV-45T were enumerated by flow cytometry according to Brussaard [34]. After thawing, the samples were diluted in 0.2 µm filtered TE buffer (10 mM Tris-HCl, 1 mM EDTA, pH 8.2), stained with the nucleic acid-specific green fluorescent dye SYBR-green I (10^−5^ dilution of commercial stock; ThermoFisher Scientific, Waltham, EUA) and incubated in the dark for 10 min at 80 °C. The samples were analyzed using a Beckton Dickinson FACS Calibur flow cytometer equipped with a 488 nm argon laser and the trigger set on GFL. MpoV-45T was discriminated using the SSC and GFL signals [27]. The latent period was defined as the time span between infection (*T* = 0) and the moment where an increase of ≥5% in viral counts was detected. The maximum virus production rate was calculated by fitting a linear regression over the period of steepest increase. MpoV-45T burst size (number of produced viruses released per lysed host cell) was calculated by dividing the maximum increase in MpoV-45T abundance (by flow cytometry) by the maximum decline in algal counts.

### 2.5. Statistical Analyses

Two-way ANOVA was used to test for the statistical difference between treatments (light intensity, light cycle, temperature, and host strain) and host growth characteristics prior to infection (i.e., maximum growth rate, Fv/Fm, FSC, SSC, RFL, and ROS), as well as between treatments and infection dynamics (time till drop in host Fv/Fm, start of lysis and full lysis of host culture, MpoV-45T latent period, maximum production rate and burst size). The host cellular characteristics were averaged over first 25 h sampling period (5 h sampling interval) to account for diel cycle variation. To further resolve statistical differences between LL, ML, and HL, Tukey’s tests were performed on the two-way ANOVA results. A probability of *p* < 0.05 was taken to conclude that the treatment differed significantly in its effect on the measured value.

## 3. Results

### 3.1. Non-Infected Controls

Under the standard culture conditions (16:8 h L:D, 3 °C), *M. polaris* strains RCC2257 and RCC2258 displayed a maximum growth rate of 0.48 d^−1^ (Table 1). HL did not affect the maximum growth rate (*p* > 0.05), while LL resulted in strongly reduced growth (0.06–0.09 d^−1^; Table 1 and Table S1). A prolonged light period (24:0 h L:D) increased the maximum growth rates of RCC2258 by 78, 21 and 22% for LL, ML and HL respectively (0.16, 0.58, and 0.60 d^−1^; Table 1 and Table S1). Similarly, a higher temperature increased the maximum growth rates for ML and HL to 0.70 and 0.69 d^−1^ (Table 1 and Table S1). All the exponentially growing cultures displayed optimal photosynthetic efficiencies with Fv/Fm between 0.61–0.69, independent of treatment (Table 1). The LL cells had the lowest average FSC (indicative of cell size [36,37]) for both algal strains (Table 1 and Table S1). Contrary to FSC, SSC did not differ markedly between treatments and strains. LL cells did show the highest average RFL (about 2 and 3-fold higher than ML and HL, respectively, Table 1 and Table S1), with strain RCC228 cells displaying overall higher RFL signals than RCC2257 (Table 1). The percentages of dead cells were never higher than 4.5% (Table 1).

**Figure 1:**
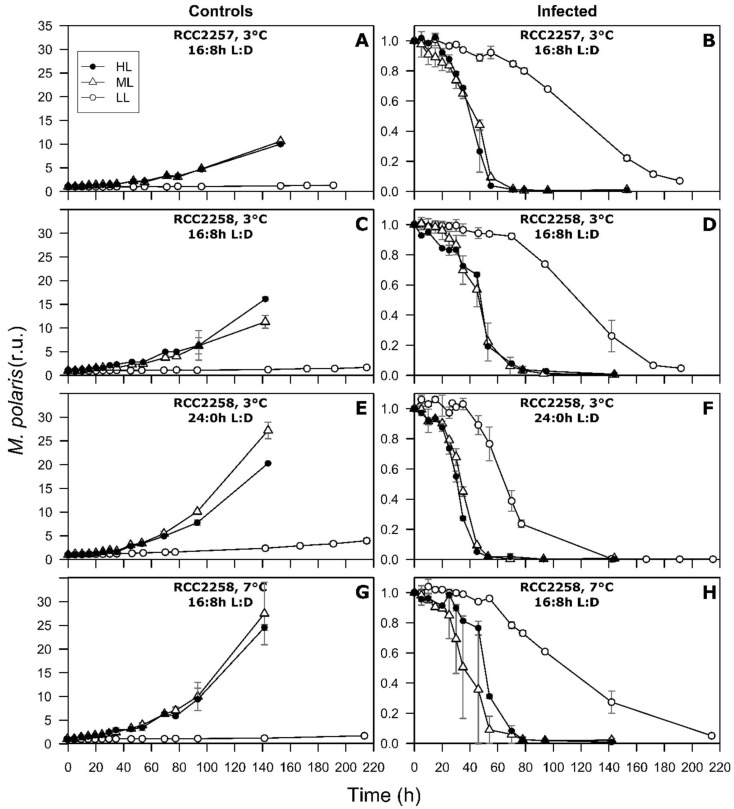
Temporal dynamics of algal abundances (in r.u., normalized to the initial abundance) for non-infected controls and MpoV-45T infected *Micromonas polaris* RCC2257 (**A**,**B**) and RCC2258 (**C**–**H**), cultured at 3 °C (**A**–**F**) and 7 °C (**G**,**H**), with a 16:8 h (**A**–**D**, **G**,**H**) and 24:0 h (**E**,**F**) light:dark (L:D) cycle and different light intensities (LL, ML and HL, i.e., respectively, 5, 60 and 160 µmol quanta m^−2^ s^−1^). LL at 3 °C and 16:8 h L:D cycle and ML at 7 °C and 16:8 h L:D have *n* = 4, ML at 3 °C and 16:8 h L:D has *n* = 6, all the other conditions have *n* = 2. Error bars represent standard deviation and r.u. stands for relative units.

**Figure 2:**
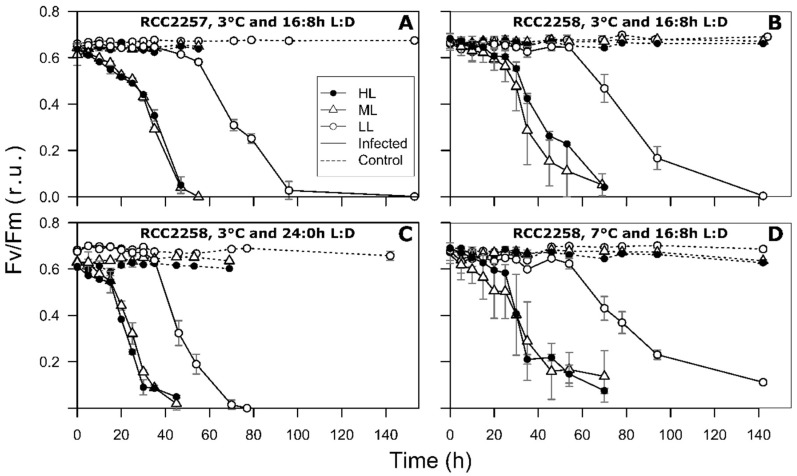
Temporal dynamics of photosynthetic efficiency (Fv/Fm) for non-infected controls (dashed lines) and MpoV-45T infected *Micromonas polaris* RCC2257 (**A**) and RCC2258 (**B**–**D**), cultured at 3 °C (**A**–**C**) and 7 °C (**D**), with a 16:8 h (**A**,**B**,**D**) and 24:0 h (**C**) light:dark (L:D) cycle and different light intensities (LL, ML and HL, i.e., respectively, 5, 60, and 160 µmol quanta m^−2^ s^−1^). LL at 3 °C and 16:8 h L:D cycle and ML at 7 °C and 16:8 h L:D have *n* = 4, ML at 3 °C and 16:8 h L:D has *n* = 6, all the other conditions have *n* = 2. Error bars represent standard deviation and r.u. stands for relative units.

**Figure 3:**
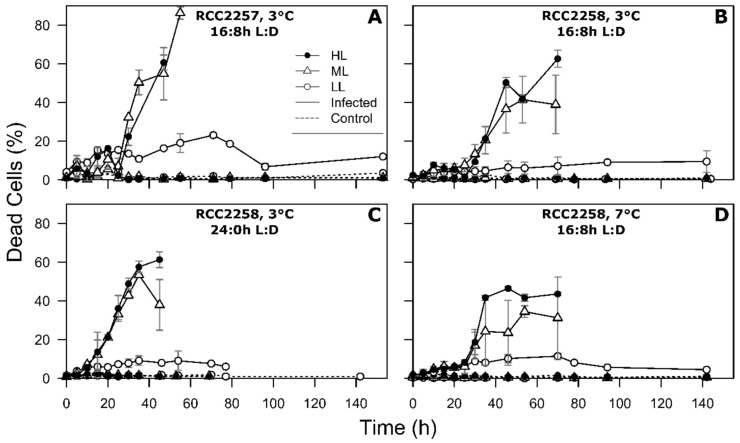
Temporal dynamics of percentage of dead cells for non-infected controls (dashed lines) and MpoV-45T infected *Micromonas polaris* RCC2257 (**A**) and RCC2258 (**B**–**D**), cultured at 3 °C (**A**-**C**) and 7 °C (**D**), with a 16:8 h (**A**, **B**, **D**) and 24:0 h (**C**) light:dark (L:D) cycle and different light intensities (LL, ML and HL, i.e., respectively, 5, 60 and 160 µmol quanta m^−2^ s^−1^). LL at 3 °C and 16:8 h L:D cycle and ML at 7 °C and 16:8 h L:D have *n* = 4, ML at 3 °C and 16:8 h L:D has *n* = 6, all the other conditions have *n* = 2. Error bars represent standard deviation and r.u. stands for relative units.

As expected, the non-infected control cultures showed continuous growth during the whole experiment (Figure 1). The control cultures showed optimal photosynthesis, with Fv/Fm between 0.61 and 0.70 (Figure 2). This was further supported by sustained low percentages of dead cells in the control cultures (Figure 3).

### 3.2. Infection Dynamics at Different Light Intensities

The HL and ML treatments exhibited very similar infection dynamics for host cell abundances, Fv/Fm, cellular characteristics (FSC, SSC, and RFL) and percentage of dead cells (Figure 1, Figure 2 and Figure 3 and Figures S1–S3), as confirmed by statistical analysis (Tukey’s test, *p* > 0.05). The initial drop in Fv/Fm of the 16:8 h L:D infected host cultures (3 °C) was within the first 24 h post infection (p.i.), however 10 to 15 h delayed for RCC2258 as compared to RCC2257 (Figure 2, Table S2). Viral infection halted the diel dynamics of the cellular characteristics 15 h p.i. (for both host strains; Figures S1–S3). Compared to the controls, FSC and SSC of the infected cells started to increase rapidly (within 12 p.i.) to a maximum 1.1 to 1.3-fold in the first 36–40 h p.i. Full lysis of the ML and HL cultures (16:8 h L:D, 3 °C) occurred within 71 h p.i. for RCC2257 and 78 h for RCC2258 (Figure 1). The percentage of dead cells (still high RFL but with compromised cell membrane) in the infected cultures confirmed that RCC2257 lysed faster than RCC2258, displaying a respective peak percentage of 86 and 42% at around 55 h p.i. (Figure 3). Cellular ROS production reduced upon host cell lysis but without a clear effect of light intensity (Figure S4).

**Figure 4:**
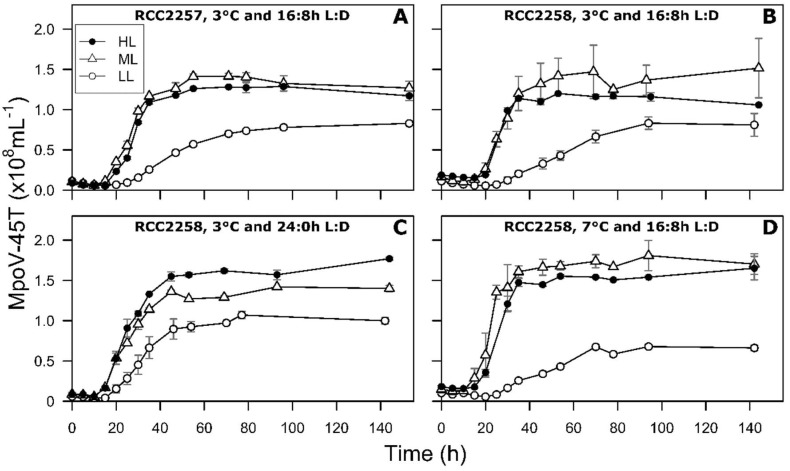
Temporal dynamics of MpoV-45T abundances in infected cultures of *Micromonas polaris* RCC2257 (**A**) and RCC2258 (**B**–**D**), cultured at 3 °C (**A**–**C**) and 7 °C (**D**), with a 16:8 h (**A**,**B**,**D**) and 24:0 h (**C**) light:dark (L:D) cycle, and under different light intensities (LL, ML and HL, i.e., respectively, 5, 60 and 160 µmol quanta m^−2^ s^−1^). LL at 3 °C and 16:8 h L:D cycle and ML at 7 °C and 16:8 h L:D have *n* = 4, ML at 3 °C and 16:8 h L:D has *n* = 6, all the other conditions have *n* = 2. Error bars represent standard deviation.

For the LL treatment under the standard light and temperature (16:8 h L:D, 3 °C) the onset of cell lysis and the drop in Fv/Fm started at least one day later than for ML and HL (Figure 1 and Figure 2, Table S2). For both host strains full lysis of the culture was reached only 8 days p.i. Notably, the percentage of dead cells in the LL infected cultures increased within a few hours p.i. but the percentages remained relatively low (max. 23 and 14% for RCC2257 and 2258; Figure 3). Nevertheless, viral progeny was released and plateaued around 4 days p.i. (Figure 4).

The latent period of MpoV-45T under the ML and HL standard culture conditions was 10–15 h for the RCC2257 cultures and 15–20 h for RCC2258 (Figure 4, Table 2). In contrast, LL prolonged the latent period for 10 and 5 h for RCC2257 and RCC2258 respectively (Table 2). The maximum virus production rate at ML was 5.6 and 5.5 × 10^6^ h^−1^ with RCC2257 and RCC2258 host respectively (Table 2). Comparable rates were found for the HL treatment (p>0.05), but at LL the rates were 3.5–5 times lower (Table 2 and Table S2). MpoV-45T burst sizes did not differ significantly for ML and HL for both host strains but were reduced by 38 and 47% at LL for RCC2258 and RCC2257 respectively (125 and 146 MpoV-45T lysed host cell^−1^, Table 2 and Table S2). The percentage of infective progeny viruses under ML (for host RCC228) were >76% while LL resulted in reduced infectivity (13–51%).

### 3.3. Effects of Light Period on Viral Infection Dynamics

The continuous light treatment (RCC228, 24:0 h L:D, 3 °C) resulted in faster infection dynamics for all three light intensities, and particularly LL (Figure 1, Figure 2 and Figure 3, Table 2 and Table S2). Again, the ML and HL treatments presented very similar infection dynamics (Table 2 and Table S2). Fv/Fm reached low values about 10 h earlier than under the 16:8 h L:D cycle (Figure 2, Table S2). Average cellular FSC and SSC still increased after infection (comparable to values at 16:8 h L:D) but showed a faster decline (within 24 h p.i.) concomitant with host cell lysis (Figures S1 and S2). The percentage of dead host cells also increased faster (Figure 3) and full lysis of the cultures (Figure 1, Table S2) occurred about 24 h earlier (within two days p.i.).

The effect of continuous light was most pronounced for the LL infection dynamics. Fv/Fm, FSC and SSC started to decrease already 35 h p.i., which is 18 h earlier than under 16:8 h L:D (Figure 2, Figures S1 and S2, Table S2). Cell lysis occurred earlier and faster, reaching full lysis 2–3 days earlier (than 16:8 h L:D; Figure 1, Table S2). The latent period of MpoV-4T was shortened by 5 h (10–15 h, Table 2), independent of light intensity. However, maximum virus production and burst size were only affected at LL, i.e., 2.6 times faster and 1.7-fold higher than under a 16:8 h L:D, respectively (Table 2). Similar to the 16:8 h L:D culture condition, the produced lysates resulting from LL cultures displayed low infectivity (9%) while infectivity at continuous ML was not affected.

### 3.4. Effect of Temperature on Viral Infection Dynamics

Cell lysis of the LL cultures at 7 °C started 15 h earlier (54 p.i.) than at 3 °C, but no significant differences in latent period, maximum virus production rate or burst size were observed (Table 2 and Table S2). In contrast, the latent period of MpoV-45T in the ML and HL treatment was 5 h shorter at 7 than at 3 °C (Table 2). Virus production rates were 1.3 and 1.2-fold higher for ML and HL at 7 °C than at 3 °C. The burst size for ML and HL was also higher at 7 than at 3 °C (Table 2 and Table S2), being 270 ± 7 and 275 ± 2, respectively. The infectivity of the progeny resulting from ML at 7 °C was strongly reduced (down to 4%) while from LL at 7 °C it remained comparably low (7 and 9% at 7 and 3 °C, respectively).

The ML 7 °C treatment showed in general large standard deviations in host lysis dynamics (Figure 1 and Figure 2, Figures S1–S3), which was due to variation in the two independent experiments performed (Figure S5). The only difference between these experiments was the type of lights used, i.e., the second experiment was executed using LED light instead of the standard fluorescent lights used for the other experiments (earlier checks displayed no difference in exponential algal growth rates). Following these results, we tested the infection of an algal culture (adapted to LED light) transferred to fluorescent light one day prior to infection, with a resulting faster lysis, comparable to the differences reported above. Note that the observed variation did not affect the virus production characteristics.

## 4. Discussion

Our results show that irradiance and temperature strongly affect interactions between *M. polaris* and MpoV-45T. Reduced light intensity (LL) prolonged the latent period, lowered maximum viral production rates, and subsequently diminished the burst size under all the temperature and light period conditions tested for both host strains. Moreover, continuous light resulted in faster infection dynamics for all light intensities, whereas increased temperature (7 °C) led to faster infection dynamics and increased viral proliferation for the ML and HL cultures only.

### 4.1. Reduced Virus Proliferation Under LL

The infection dynamics for the HL and ML cultures (160 and 60 µmol quanta m^−2^ s^−1^) were largely comparable, whereas the LL treatment displayed prolonged infection dynamics and reduced virus production. The latter was likely due to declined metabolic state (reduced energy availability resulting in lowered exponential growth rate) of the host cells [28]. The concomitantly smaller cell size (FSC) and higher cellular chlorophyll content (adaptation to improve the light harvesting capacity of the cell) supports the conclusion that these cultures were energy limited at 5 µmol quanta m^−2^ s^−1^. Picophytoplankton like *Micromonas* are relatively efficient at harvesting light at low intensities [40]. This may explain why earlier studies on the interaction of MpV with *Micromonas commoda* LAC38 at 25 µmol quanta m^−2^ s^−1^ did not observe an extended latent period and reduced burst size [29,30]. The very low light intensity used in our study is ecologically relevant to the Arctic [41,42], and hence the strong negative impact it had on virus proliferation observed could account in the natural environment for sustained host occurrence at the start and end of the productive season as well as throughout the season under the ice near the edges of the ice shelf (low light conditions). Additionally, the slower virus proliferation may act as a mechanism to avoid its annihilation by host extinction in a period where the recovery of the host population will be slow.

Alternatively, a prolongation of the lytic cycle under LL could be beneficial, as more time from infection to lysis would mean that more energy for viral proliferation could be freshly harvested by the chloroplast. Besides, as observed in this study for LL, infected strains of *Micromonas* showed prolonged chloroplast integrity and maintenance of high photosynthetic efficiency under non-optimal host growth rates [43,44]. Such conservation of an efficient photosynthetic system (maintaining optimal Fv/Fm under LL) may result from an active maintenance carried by viral auxiliary metabolic genes as observed for many cyanophages [45]. Increased proliferation of bacteriophage T4 (infecting *Escherichia coli*) [46,47] through an extended latent period has indeed been shown to be an effective way for the phage to optimize proliferation under suboptimal conditions.

Both *M. polaris* strains RCC2257 and 2258 showed similar virus growth dynamics under LL, but under ML and HL the latent period of MpoV-45T was 5 h shorter when infecting strain RCC2257. Since both strains were isolated together (adjacent stations in the Beaufort Sea during August 2009, 1 day apart), the differences in response to viral infection probably illustrates natural variation. Maat et al. [27] documented comparable host strain-specific virus growth characteristics (latent period and burst size) upon infection of different *M. polaris* strains (with MpoV-45T at ML) originating from different geographical locations and isolated in different years. Light-induced variability in host strain response to infection may thus contribute to intraspecies diversity.

The LL cultures showed a pronounced mismatch between the increase of extracellular virus progeny and host lysis (overall low percentage dead cells while many cells still with intact RFL). The underlying mechanism is currently unclear but it could be that (i) the cell membrane of LL *M. polaris* was not compromised (low number of SYTOX stained cells) to the same extent as ML/HL, or (ii) the longer virus production period led to a longer host lysis period, whereby host cell membranes may be compromised more slowly but then lysis was as fast as under ML and HL, or (iii) there was a higher turnover of dead cells (faster lysis of stainable cells). Again, the LL cells responded differently in their cellular characteristics upon infection compared to ML and HL by not increasing their FSC and SSC. The increased cellular FSC and SSC, indicative of larger cells with increased granularity, of infected host at ML and HL may be due to accumulation of viral particles in the host’s cytoplasm (*M. pusilla*, [48]) or necrotic swelling prior to lysis [49]. Possibly correlated to the latter is the lack of buildup of cellular ROS in infected *M. polaris*, as has been reported for virally infected *Emiliania huxleyi* in relation to programmed cell death [39,50,51]. We even noted (for all light intensities) a reduction in ROS upon infection in the light period when the diel ROS production in the non-infected control cells was at its highest (probably related to growth regulatory and signaling processes [52,53]. The swollen infected cells at ML and HL may potentially increase their edibility or availability for larger zooplankton [54], e.g. in the surface ocean or at the end of spring and summer. Besides, changes in the speed of host lysis after infection can have significant ecological implications. Under faster lysis, as observed under continuous light or at 7 °C (Section 4.2 and Section 4.3), the time frame of infected prey availability for zooplankton grazing would be shorter and thus fostering the viral shunt.

### 4.2. Continuous Light Enhances Virus Proliferation

Host production at LL was particularly stimulated by the increased total amount of photons under continuous light as compared to similar light intensities under 16:8 h L:D, resulting in a 1.8-fold increase in growth rate (vs 1.2-fold for ML and HL). This resulted in faster infection dynamics with shorter latent period and faster lysis, (compared to a 16:8 h L:D regime). Shortening of the latent period under ML seems a more general feature as it was also observed for MpoV-45T infecting another host strain *M. polaris* TX-01 (from 15–18 h at 16:8 h L:D to 12–16 h at continuous light, unpublished data). For both host strains (RCC2258 and TX-01) prolonged light did not significantly change virus burst size for ML and HL, yet the faster dynamics will stimulate a faster spread of the infection through the host population. Continuous LL not only resulted in faster infection dynamics but also 2.6-fold higher production rate and 1.7-fold higher burst size. Although virus infectivity at continuous LL light remained low (i.e., only 9% infective), still more infective viruses are produced (higher burst sizes), implying that the LL-induced reduction in MpoV-45T proliferation can be partly mitigated by continuous light (e.g. at depth during the Arctic summer).

### 4.3. MpoV-45T Propagation is Temperature Dependent

For the ML and HL cultures, increased temperature resulted in faster infection dynamics and higher burst sizes. Maat et al. [27] also reported a shortened latent period for MpoV-45T at higher temperature (and ML). However, this was only observed for host strain RCC2257 and not for host strains TX-01 or RCC2258. Cultivation conditions for RCC2258 were similar between both studies but ongoing adaptation of RCC2258 to 7 °C (selecting for cells capable of allowing faster virus proliferation) cannot be ruled out. The stimulating effects of enhanced temperature were not observed for LL, indicating that light limitation was regulating virus proliferation more than the temperature.

The enhanced MpoV-45T proliferation at higher temperature may (partly) compensate for the reduced infectivity at 7 °C. Sensitivity to temperature can be highly virus-specific, as also shown for four Artic *M. polaris* virus isolates [27]. When exposed to temperatures between 0 and 7 °C, infectivity responses of the different virus isolates varied from no effect to highest infectivity at 3 or 0 °C (i.e., MpoV-45T). Our results, in combination with those previously reported, demonstrate that it is likely that MpoV-45T will be most effective at controlling host population dynamics during early summer when temperature is still round 3 °C and light intensity and day length are increasing. In a future warming Arctic, the sea surface temperature will reach 7 °C earlier in the season [13,55] and MpoV-45T dominance is likely lessened in favor of other MpoVs that can withstand higher temperatures [27]. Considering the longer day time during the Arctic summer, we recommend future studies to elucidate the potential co-effects of temperature and light period for different host–virus model systems.

### 4.4. Ecological Implications

Differences in virus growth characteristics from naturally shifting environmental factors affect the rate at which viruses propagate through host populations, thereby affecting pelagic food web dynamics [8]. Shorter latent periods and higher maximum viral production rates, as observed under increased temperature (7 °C) and day length (24:0 h L:D) for ML and HL cultures, will lead to increased propagation of viral infection. This will result in a stronger viral top-down control of the host population, shunting more energy and organic matter towards the microbial loop and away from higher trophic levels [8].

**Figure 5:**
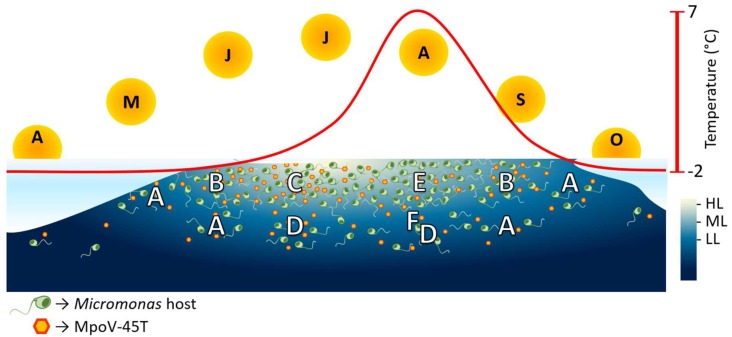
The predicted response of *M. polaris* host and MpoV-45T populations during the arctic productive season. The letters inside the symbolic suns represent the months (April to October) and the red line represent the change in temperature of the surface ocean along the season. The symbols in the water column represent the predicted spatiotemporal dynamics of the algal host and its virus MpoV-45T under influence of irradiance and temperature (based on our results). The white letters in the water column represent the possible spatiotemporal occurrences of the conditions referred to in the discussion (A–F). LL, ML, and HL stand for low, mid and high light intensity (5, 60, and 160 µmol quanta m^−2^ s^−1^).

In Figure 5 we synthesize our results and project the current consequences of seasonal changes in light (intensity and day length) and temperature in Arctic waters on the temporal dynamics of this picoeukaryotic host and its virus. Low light conditions dominate the beginning and the end of the Arctic productive season due to the low altitude of the sun, increased wind mixing and ice and snow cover. The prevailing LL condition will strongly reduce virus infectivity and virus proliferation after successful infection will take days. With the increasing light during early spring the low growth of *M. polaris* will rise and its abundance will increase over time (condition A, Figure 5). With continued increasing light intensity, host growth and virus infectivity improve, as does virus proliferation and speed of host lysis (condition B). Higher light intensities during spring will not affect virus production or host growth any further, but the concomitantly lengthening light period and slowly increasing temperature in the surface waters will lead to an even faster spread of infection through the host population (condition C). LL condition can still be found deeper in the water column, but the long daylight provides more energy and as such, higher burst sizes (condition D). With the progressing warming and subsequent ice melt, a salinity-induced vertical stratification of the water column will develop. Although Arctic *Micromonas* has been reported to grow well at relatively lower salinities [23,56], it is still unknown what effect salinity may have on the virus infection characteristics. The relatively warm seawater and long day length will further increase the host growth rate [this study, 23]. Proliferation of MpoV-45T is fast in these warmer waters, yet its progeny will rapidly lose infectivity (condition E). Nutrient depletion during summer will favor picoeukaryotic species like *M. polaris* [18,19,22,23,26], however it is likely that virus proliferation is still negatively impacted [30,57,58]. Deeper in the stratified water column during summer, continuous LL conditions prevail which will potentially further prolong the latent period and reduce virus production (condition F). Slightly deeper, the temperature drops (condition D) while nutrient limitation will decrease; as such virus proliferation will be stimulated compared to the condition F. Nonetheless, LL will render most of the MpoV-45T progeny non-infective, thereby reducing the importance of viral lysis as loss factor and promoting survival of the host at those depths. From late summer to autumn, as surface temperature drops and days get shorter, host growth and MpoV-45T proliferation reduces (longer latent period, reduced burst size and prolonged host lysis; condition B). Yet, the temperature-induced reduction in infectivity is diminished and so viral control of the host population will be enforced. Finally, the autumn to winter transition will be mainly dominated by lowered temperature and light availability (condition A), as in early spring, reducing viral impact again in response to the low light. Under dark conditions, infection of other *Micromonas* species is reported to halt [29,43,59,60], yet, *M. polaris* may potentially survive the winter as a mixotroph [61,62]. If so, it might be that it has enough energy to produce viruses upon infection, presenting an alternative explanation for the low abundances of *Micromonas* and high abundances of its viruses in Arctic winter metagenomes [63]. The present study provides new insight on how key environmental variables in the Arctic control the interactions between the picophytoplankter *M. polaris* and its lytic virus during the productive season. Studying the mechanisms underlying the survival of the virus and its host through the polar winter will help to understand the mechanisms underlaying the onset of the productive season.

## Figures and Tables

**Table 1:** Exponential growth rates (Growth, d^−1^), photosynthetic efficiency (Fv/Fm, r.u.), mean cellular forward and side scatter (FSC and SSC, r.u.), red Chlorophyll autofluorescence (RFL, r.u.), cellular reactive oxygen species Content (ROS, r.u.) and dead cells (%) of *Micromonas polaris* RCC2258 and RCC2257 adapted to an incubation temperature (Temp) of 3 or 7 °C, a 16:8 h or 24:0 h light:dark cycle and at a light intensity (Light Int.) of 5 (LL), 60 (ML) or 160 (HL) µmol quanta m^−2^ s^−1^. Cultures under 16:8 h light condition displayed synchronized cell division and therefore cellular characteristics were averages over a 24 h diel cycle. R.u. stands for relative units, NA stands for not available.

Host Strain	Temp	Light Cycle	Light Int.	Growth	Fv/Fm	FSC	SSC	RFL	ROS	Dead Cells
RCC2257	3	16:8	LL	0.06	0.67	414	23	200	77	2.2
			ML	0.48	0.64	597	25	96	54	1.1
			HL	0.49	0.64	679	23	65	59	1.1
RCC2258	3	16:8	LL	0.09	0.66	458	23	381	76	0.5
			ML	0.48	0.67	735	27	177	50	0.7
			HL	0.49	0.67	789	27	136	NA	1.4
RCC2258	3	24:0	LL	0.16	0.69	550	24	317	NA	1.4
			ML	0.58	0.64	892	29	144	NA	1.5
			HL	0.60	0.61	955	28	108	NA	1.5
RCC2258	7	16:8	LL	0.11	0.67	571	25	330	49	0.3
			ML	0.70	0.67	771	27	148	61	0.5
			HL	0.69	0.67	797	27	137	NA	1.6

**Table 2:** Virus growth characteristics, i.e., latent period (h), maximum production rate (Max. Prod., ×10^6^ h^−1^), and burst size (total produced viruses lysed host cell^−1^) of MpV-45T infecting *M. polaris* RCC2258 and RCC2257 cultured at two temperatures (Temp., 3 °C and 7 °C), under a 16:8 h and 24:0 h light:dark cycle at low (LL), mid (ML) and high (HL) light intensity.

Host Strain	Temp.	Light Cycle	Light Intensity	Latent Period	Max. Prod.	Burst Size
			LL	20–25	1.6 ± 0.0	146 ± 1
RCC2257	3	16:8	ML	10–15	5.6 ± 0.3	237 ± 4
			HL	10–15	5.3 ± 0.1	251 ± 5
			LL	20–25	1.4 ± 0.2	125 ± 8
RCC2258	3	16:8	ML	15–20	5.5 ± 0.5	235 ± 44
			HL	15–20	6.6 ± 0.0	214 ± 16
			LL	15–20	3.6 ± 0.3	211 ± 1
RCC2258	3	24:0	ML	10–15	5.1 ± 2.0	238 ± 6
			HL	10–15	5.8 ± 0.0	250 ± 2
			LL	20–25	1.3 ± 0.0	137 ± 15
RCC2258	7	16:8	ML	10–15	8.3 ± 1.6	270 ± 7
			HL	10–15	7.6 ± 0.1	275 ± 2

## Supplementary Materials

The following are available online at http://www.mdpi.com/1999-4915/10/12/676/s1, Figure S1: Temporal dynamics of cellular forward scatter of non-infected controls and MpoV-4T infected *Micromonas polaris*, Figure S2: Temporal dynamics of cellular side scatter of non-infected controls and MpoV-4T infected *Micromonas polaris*, Figure S3: Temporal dynamics of cellular chlorophyll red autofluorescence of non-infected controls and MpoV-4T infected *Micromonas polaris*, Figure S4: Temporal dynamics of cellular reactive oxygen species of non-infected controls and MpoV-4T infected *Micromonas polaris*. Figure S5: Temporal dynamics, during two independent experiments, of algal abundances, photosynthetic efficiency, cellular characteristics, percentage of dead cells and MpoV-45T abundances for MpoV-45T infected *Micromonas polaris* RCC2258 cultured at 7 °C with a 16:8 h L:D and under ML light intensity (60 µmol quanta m^−2^ s^−1^). Table S1: Statistical two-way ANOVA of treatments with interaction terms affecting M. polaris growth and cellular characteristics. Table S2: Statistical two-way ANOVA of treatments with interaction terms affecting infection dynamics for M. polaris host and MpoV-45T.
