# Supplementary material for: Influence of Irradiance and Temperature on the Virus MpoV-45T Infecting the Arctic Picophytoplankter Micromonas polaris
Source: Viruses. 2018 Nov 29;10(12):676. doi: 10.3390/v10120676 (PMC6316886; doi:10.3390/v10120676)
Supplement: Supplementary file 1 [file viruses-10-00676-s001.pdf]

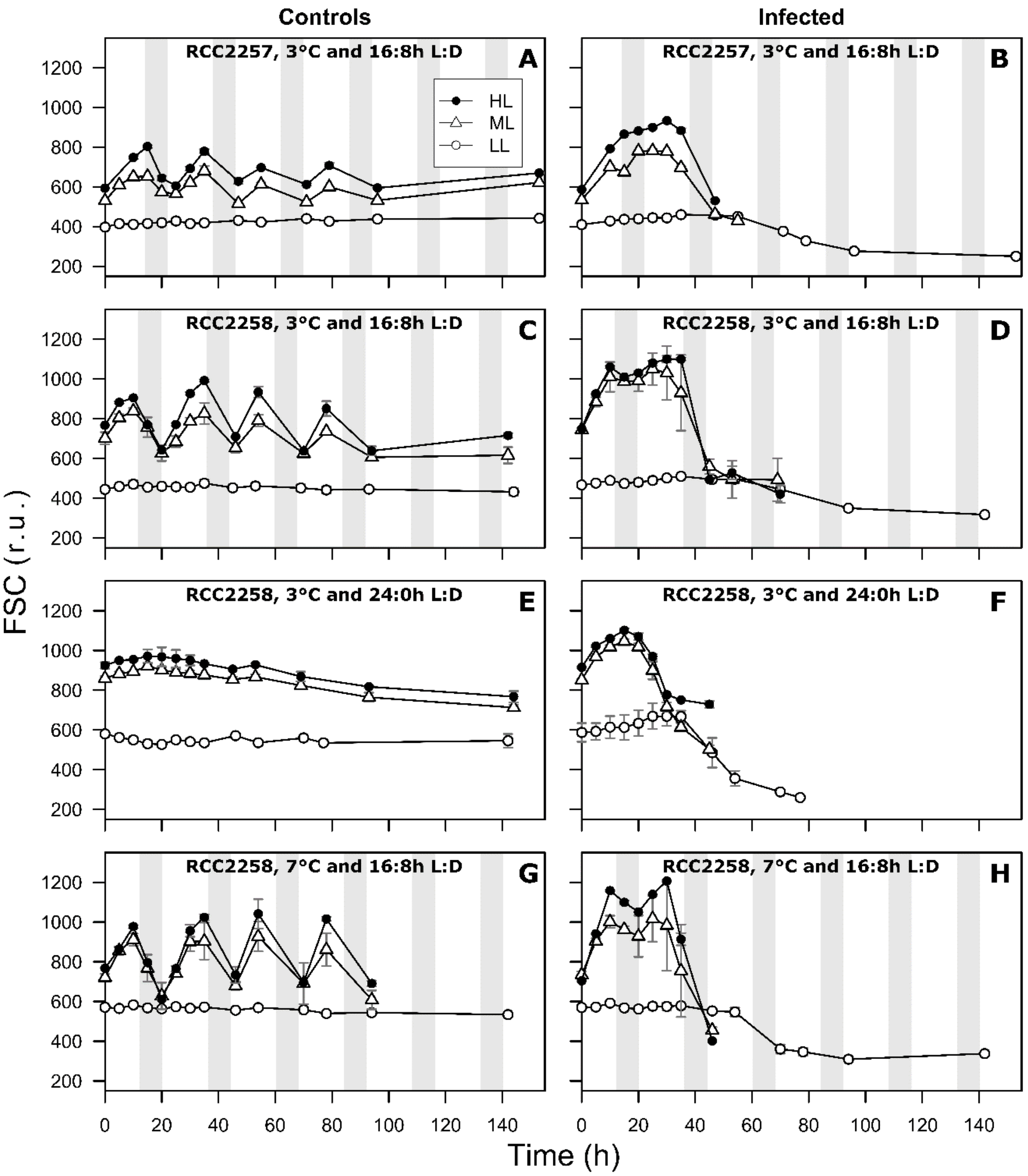

Figure S1. Temporal dynamics of cellular forward scatter (FSC) for non-infected controls and MpoV-45T infected *Micromonas polaris* RCC2257 (A, B) and RCC2258 (C-H), cultured at 3 °C (A-F) and 7 °C (G, H), with a 16:8 h (A-D, G, H) and 24:0 h (E,F) light:dark (L:D) cycle and different light intensities (LL, ML and HL, i.e. respectively, 5, 60 and 160 µmol quanta m^{-2} s^{-1}). The grey bars represent the 8 h dark period. LL at 3 °C and 16:8 h L:D cycle and ML at 7 °C and 16:8 h L:D have n=4, ML at 3 °C and 16:8 h L:D has n=6, all the other conditions have n=2, error bars represent standard deviation. r.u. stands for relative units. For the controls, the maximum values displayed the interphase of mitosis. During the subsequent night these cells showed a synchronized cell division that lead to a drop in the cellular signals. The continuous light (24:0 h L:D) treatments did not exhibit a distinct diel cycle due to non-synchronized growth. Viral infection halted the diel dynamics of the cellular characteristics.

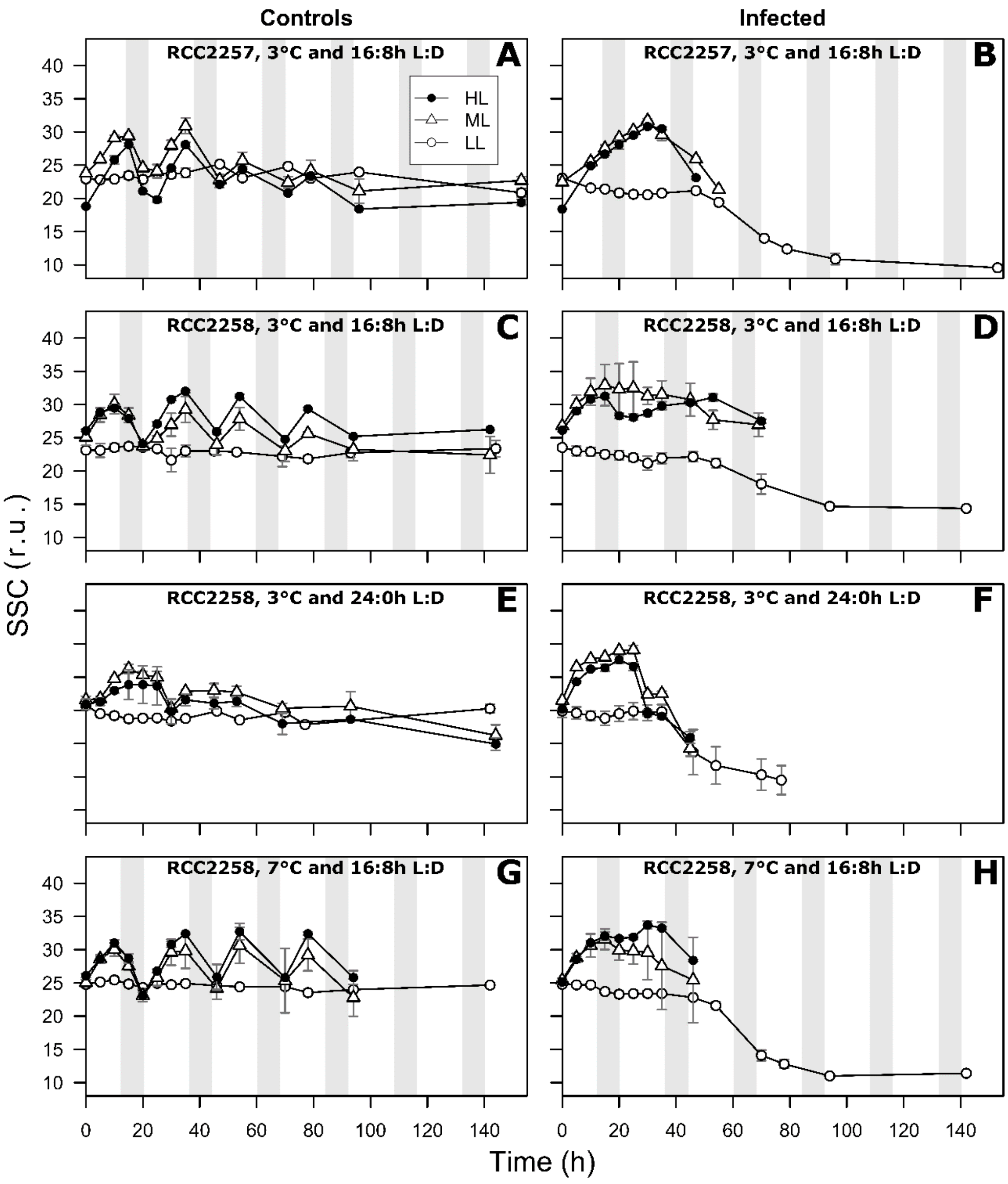

Figure S2. Temporal dynamics of cellular side scatter (SSC) for non-infected controls and MpoV-45T infected *Micromonas polaris* RCC2257 (A, B) and RCC2258 (C-H), cultured at 3 °C (A-F) and 7 °C (G, H), with a 16:8 h (A-D, G, H) and 24:0 h (E, F) light:dark (L:D) cycle and different light intensities (LL, ML and HL, i.e. respectively, 5, 60 and 160 µmol quanta m^{-2} s^{-1}). The grey bars represent the 8 h dark period. LL at 3 °C and 16:8 h L:D cycle and ML at 7 °C and 16:8 h L:D have n=4, ML at 3 °C and 16:8 h L:D has n=6, all the other conditions have n=2, error bars represent standard deviation. r.u. stands for relative units. For the controls, the maximum values displayed the interphase of mitosis. During the subsequent night these cells showed a synchronized cell division that lead to a drop in the cellular signals. The continuous light (24:0 h L:D) treatments did not exhibit a distinct diel cycle due to non-synchronized growth. Viral infection halted the diel dynamics of the cellular characteristics.

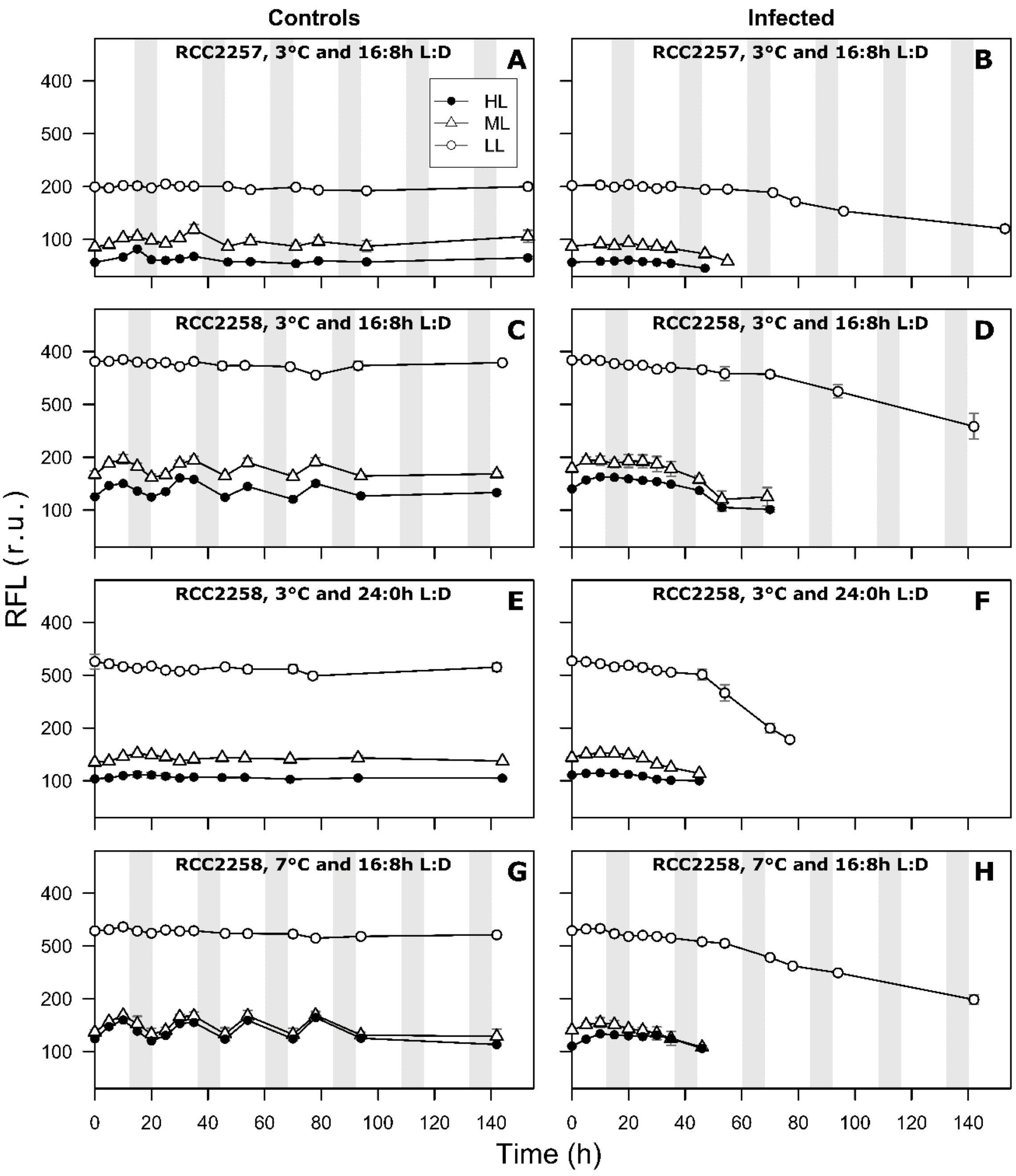

Figure S3. Temporal dynamics of cellular chlorophyll red autofluorescence (RFL) for non-infected controls and MpoV-45T infected *Micromonas polaris* RCC2257 (A, B) and RCC2258 (C-H), cultured at 3 °C (A-F) and 7 °C (G, H), with a 16:8 h (A-D, G, H) and 24:0 h (E, F) light:dark (L:D) cycle and different light intensities (LL, ML and HL, i.e. respectively, 5, 60 and 160 µmol quanta m^{-2} s^{-1}). The grey bars represent the 8 h dark period. LL at 3°C and 16:8 h L:D cycle and ML at 7 °C and 16:8 h L:D have n=4, ML at 3°C and 16:8 h L:D has n=6, all the other conditions have n=2, error bars represent standard deviation. r.u. stands for relative units. The maximum values displayed the interphase of mitosis. During the subsequent night these cells showed a synchronized cell division that lead to a drop in the cellular signals (Figure S1). The continuous light (24:0 h L:D) treatments did not exhibit a distinct diel cycle due to non-synchronized growth.

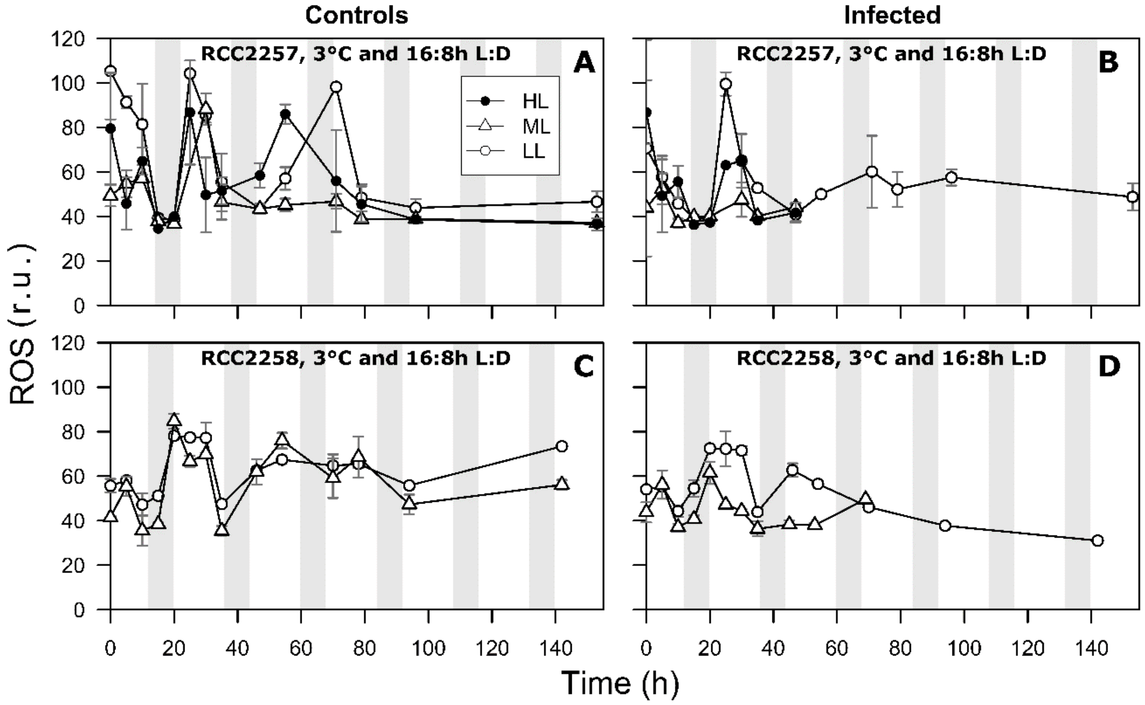

Figure S4. Temporal dynamics of cellular reactive oxygen species (ROS) green fluorescence for non-infected controls and MpoV-45T infected *Micromonas polaris* RCC2257 (A, B) and RCC2258 (C, D), cultured at 3 °C with a 16:8 h light:dark (L:D) cycle and different light intensities (LL, ML and HL, i.e. respectively, 5, 60 and 160 µmol quanta m^{-2} s^{-1}). The grey bars represent the 8 h dark period. All the conditions have n=2, error bars represent standard deviation. r.u. stands for relative units. The mean cellular ROS also showed a diel cycle with higher signals at the first light of the day, reducing during the day until it reached a minimum during the night.

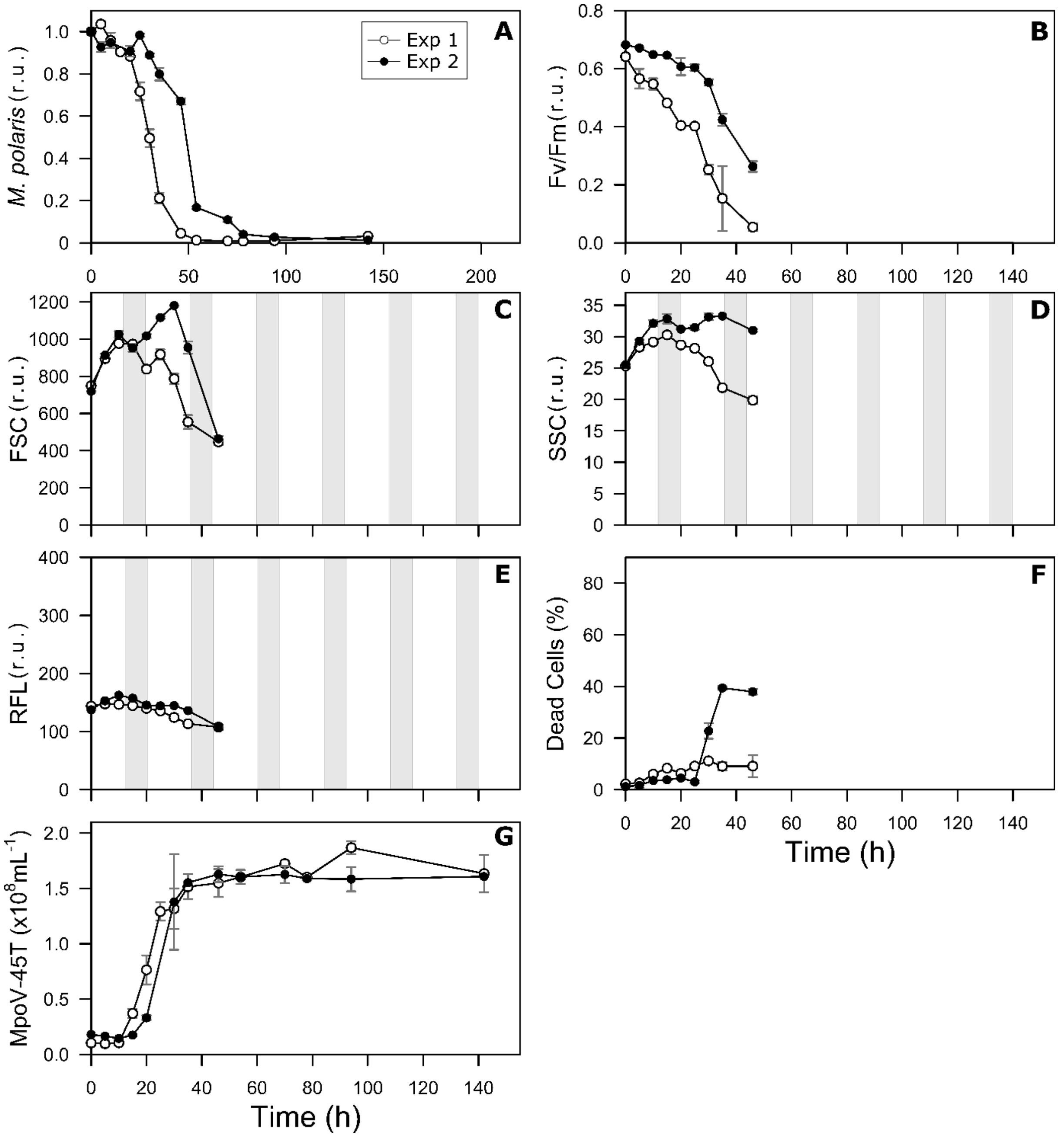

Figure S5. Temporal dynamics, during two independent experiments (Exp 1 and Exp 2), of algal abundances (A, normalized to initial abundance), Fv/Fm (B), mean cellular FSC (C), SSC (D) and RFL (E) from infected *Micromonas polaris* RCC2258 cultured at ML light intensity (60 µmol quanta m^{-2} s^{-1}), 16:8 h light:dark and 7 °C. MpoV-45T abundances were comparable for the two experimental replicates Exp 1 and Exp 2 (G). Error bars represent standard deviation and r.u. stands for relative units.

Table S1. Statistical two-way ANOVA of treatments (three levels for light intensity, two levels for light cycle, temperature and host strain) with interaction terms affecting *M. polaris* growth and cellular characteristics. The treatments that were found to significantly influence exponential growth rate, Fv/Fm, FSC, SSC, RFL, and ROS by asterisks: *, ** and *** represent P-values <0.05, <0.01 an <0.001. n.s. stands for not significant, and NA for not available.

**Host strain**	**Temp.**	**Light cycle**	**Factor**	**Growth**	**Fv/Fm**	**FSC**	**SSC**	**RFL**	**ROS**
RCC2257			Strain	***	n.s.	***	***	***	n.s.
vs	3 °C	16:8 h	Light intensity	***	n.s.	***	***	***	n.s.
RCC2258			Interaction	n.s.	n.s.	**	***	***	n.s.
		16:8 h	Light cycle	***	**	***	**	***	*NA*
RCC2258	3 °C	vs	Light intensity	***	**	***	***	***	*NA*
		24:0 h	Interaction	*	***	**	n.s.	***	*NA*
	3 °C		Temperature	***	n.s.	***	**	***	n.s.
RCC2258	vs	16:8 h	Light intensity	***	n.s.	***	***	***	*
	7 °C		Interaction	***	n.s.	**	*	***	n.s.

Table S2. Statistical two-way ANOVA of treatments (three levels for light intensity, two levels for light cycle, temperature and host strain) with interaction terms affecting infection dynamics for *M. polaris* host (drop in Fv/Fm, start of lysis and full lysis of the culture) and MpoV-45T (latent period, maximum production and burst size). The treatments that were found to be significant are indicated by asterisks**:** *, ** and *** represent P-values <0.05, <0.01 an <0.001. n.s. stands for not significant. The Tukey's test following the two-way ANOVA showed consistently that ML and HL were comparable while LL was significantly different from ML and HL (for all tests p<0.0001).

**Host strain**	**Temp.**	**Light cycle**	**Factor**	**Drop Fv/Fm**	**Lysis Start**	**Full Lysis**	**Latent Period**	**Max. Prod.**	**Burst Size**
RCC2257			Strain	**	**	n.s.	***	n.s.	n.s.
vs	3 °C	16:8 h	Light intensity	***	***	***	***	***	***
RCC2258			Interaction	**	***	**	*	*	n.s.
		16:8 h	Light cycle	***	***	***	***	*	*
RCC2258	3 °C	vs	Light intensity	***	***	***	***	***	***
		24:0 h	Interaction	n.s.	***	***	n.s	***	n.s.
	3 °C		Temperature	***	***	*	***	***	**
RCC2258	vs	16:8 h	Light intensity	***	***	***	***	***	***
	7 °C		Interaction	n.s.	**	n.s.	*	*	n.s.
